# Supplementary material for: Development of broadly neutralizing antibodies targeting the cytomegalovirus subdominant antigen gH
Source: Commun Biol. 2022 Apr 25;5:387. doi: 10.1038/s42003-022-03294-z (PMC9038728; doi:10.1038/s42003-022-03294-z)
Supplement: Supplementary file 2 — Supplementary Information [file 42003_2022_3294_MOESM2_ESM.pdf]

Supplementary Information

Development of broadly neutralizing antibodies targeting the cytomegalovirus subdominant antigen gH

Andrea J. Parsons[1], Sabrina I. Ophir[1], J. Andrew Duty[1,2], Thomas A. Kraus[2], Kathryn R. Stein[1], Thomas M. Moran[1,2], Domenico Tortorella[1,*]

[1] Department of Microbiology, Icahn School of Medicine at Mount Sinai, New York, NY 10029, USA
[2] Center of Therapeutic Antibody Development, Icahn School of Medicine at Mount Sinai, New York, NY 10029, USA

*Correspondence: domenico.tortorella@mssm.edu

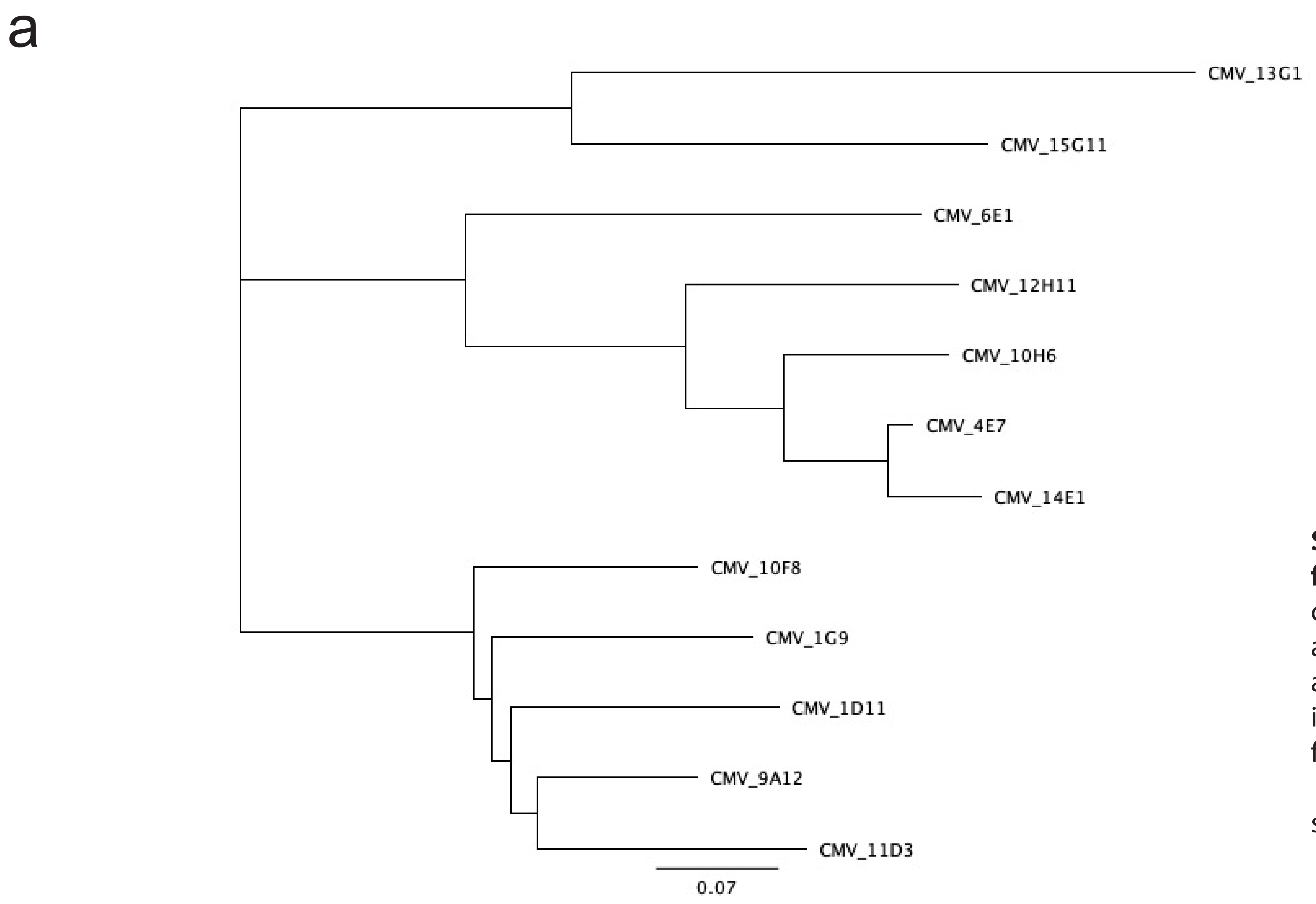

Supplementary Figure 1. Antibody CDR3 diversity and IgG allele frequency following immunization (a) A phylogenetic tree comparing heavy chain CDR3 sequences for each antibody was assembled to identify reoccurring hypersomatic mutations that arise separately in three individual mice. Antibodies can be clustered into three main branches. (b) Allelic frequency and gene usage for the V, D, J regions of the heavy chain for each antibody are provided. (c) Immunoglobulin heavy chain V, D and J gene segment summarized by gene family.

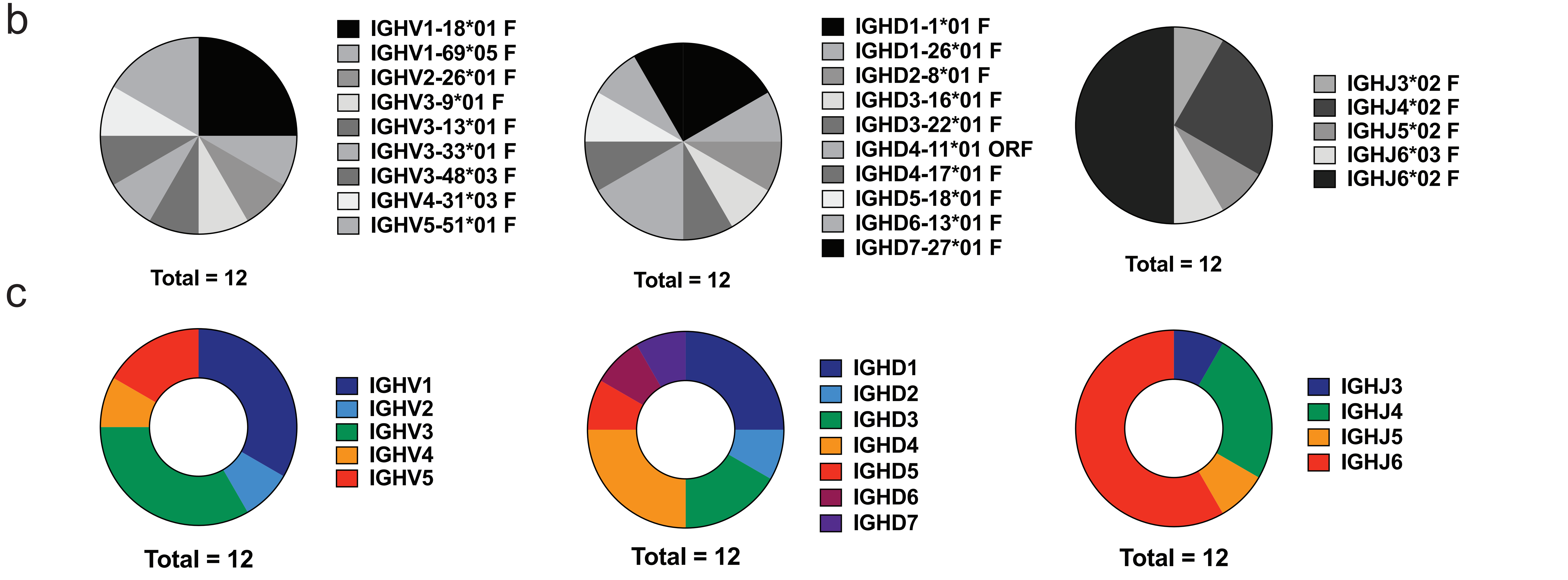

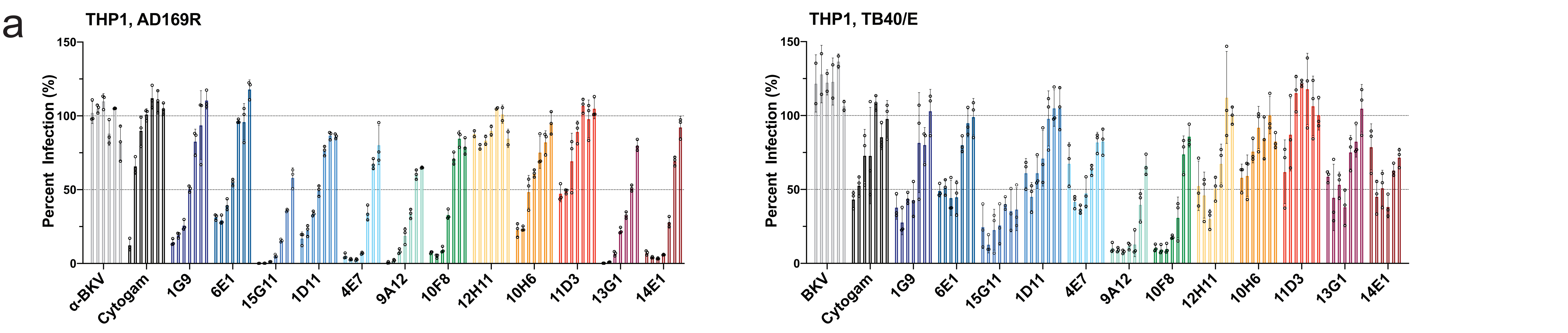

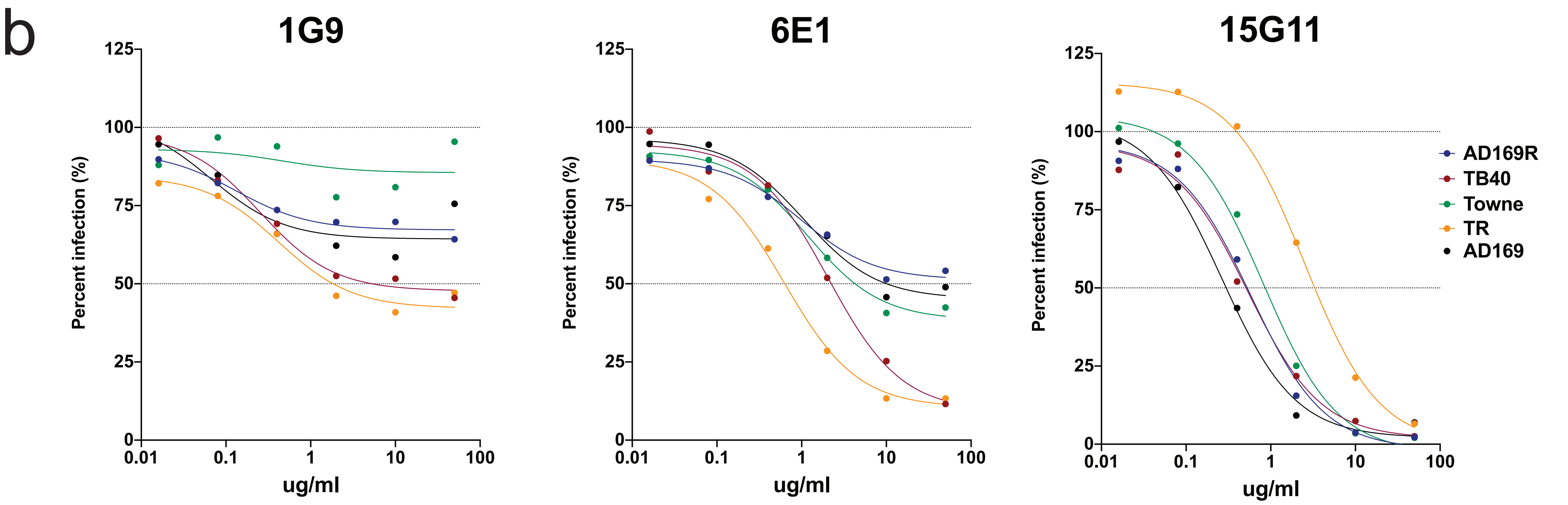

Supplementary Figure 2. Broad neutralization of HCMV gH antibodies by virus strain and cell type. (a) Neutralization in THP-1 cells for anti-gH antibodies using both AD169R (MOI 0.2) and TB40/E (MOI 0.2). (b) Neutralization curves of anti-gH antibodies against several HCMV strains in fibroblasts. Antibody (50-0.016 μg/mL, 5-fold dilutions) neutralization was assessed following infection with diverse HCMV strains (MOI 0.2). The subtle differences in neutralization capacity could be explained by genetic variation in the highly conserved gH glycoprotein.

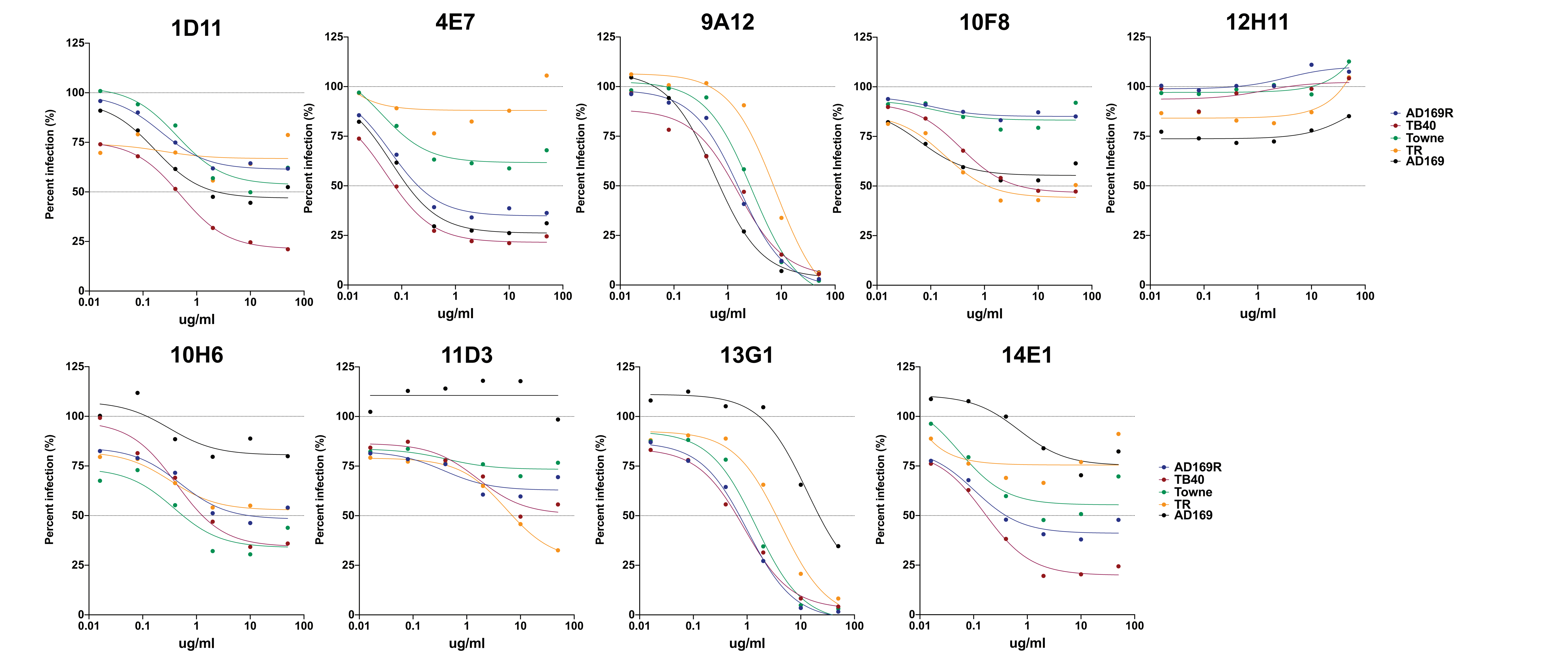

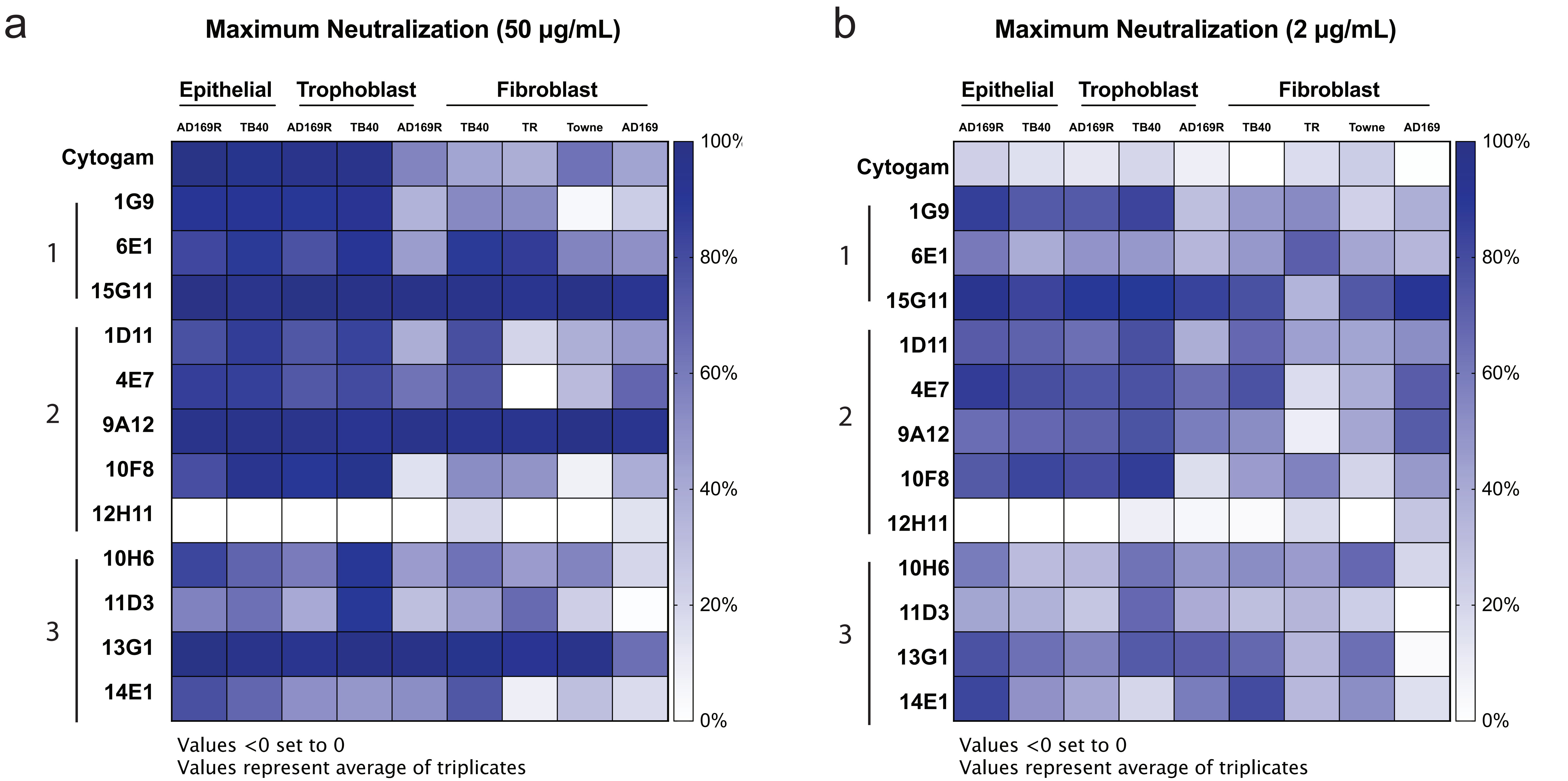

Supplementary Figure 3. Maximum neutralization of gH antibodies in comparison to Cytogam®. The percent neutralization (%) for each antibody was calculated for each condition where antibody concentration was either 50 µg/mL or 2 µg/mL. Percent neutralization was summarized in a heat map using GraphPad Prism 9 to highlight antibody efficacy at lower concentrations in comparison to Cytogam®.

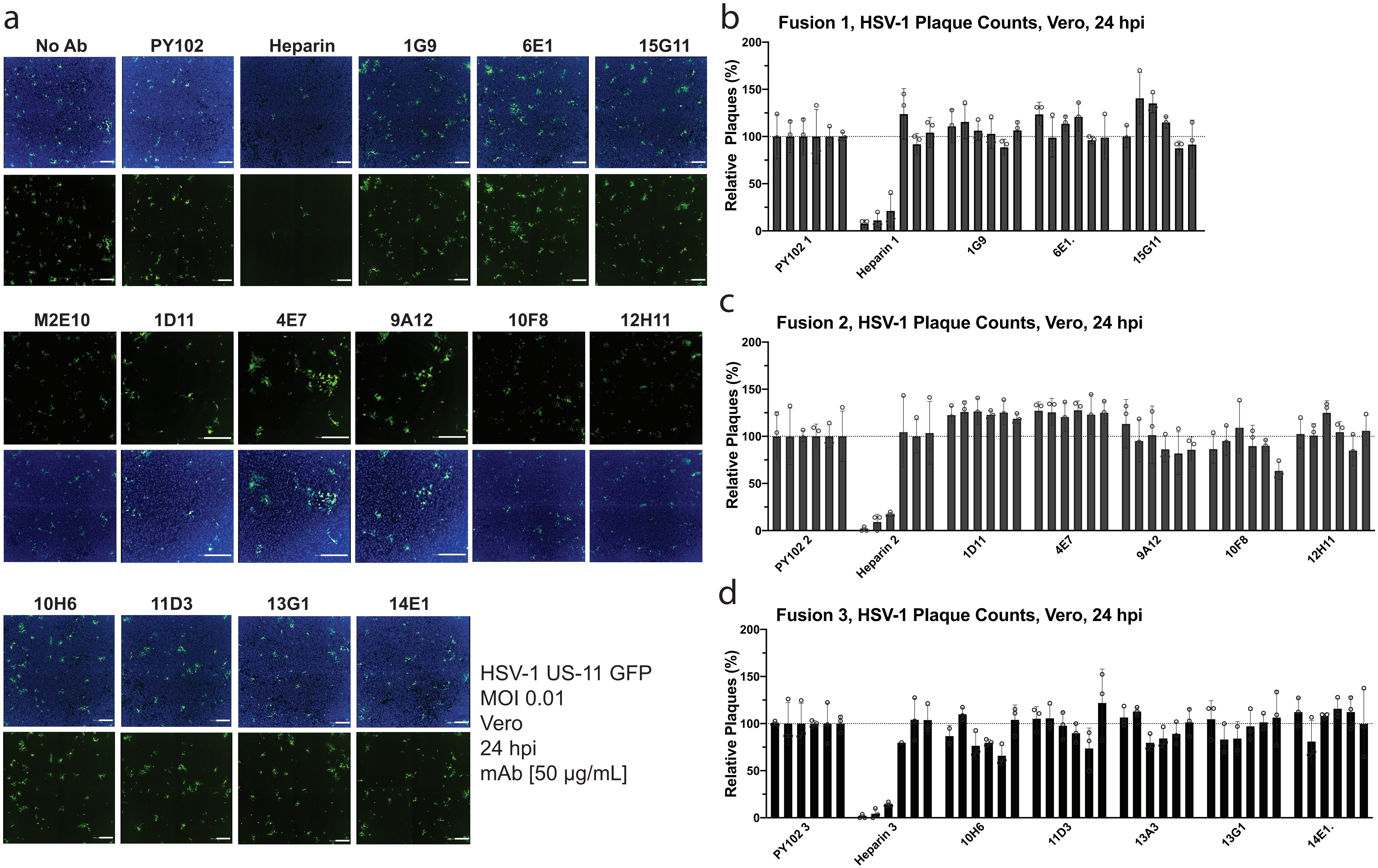

Supplementary Figure 4. gH mAbs are specific for HCMV neutralization. Neutralization of HSV-1 US-11 GFP (MOI 0.01) using anti-gH mAbs (50-0.016 μg/mL) and assessed using GFP as a readout for infection at 24 hpi in Vero cells. Representative images for each condition are shown (a) and the relative number of plaques per condition at 24 hpi are presented relative to HSV-1 neutralization by the irrelevant influenza antibody PY102 (b). Heparin (50-0.016 μg/mL) was used as a positive control for each plate and each condition was done in triplicate.

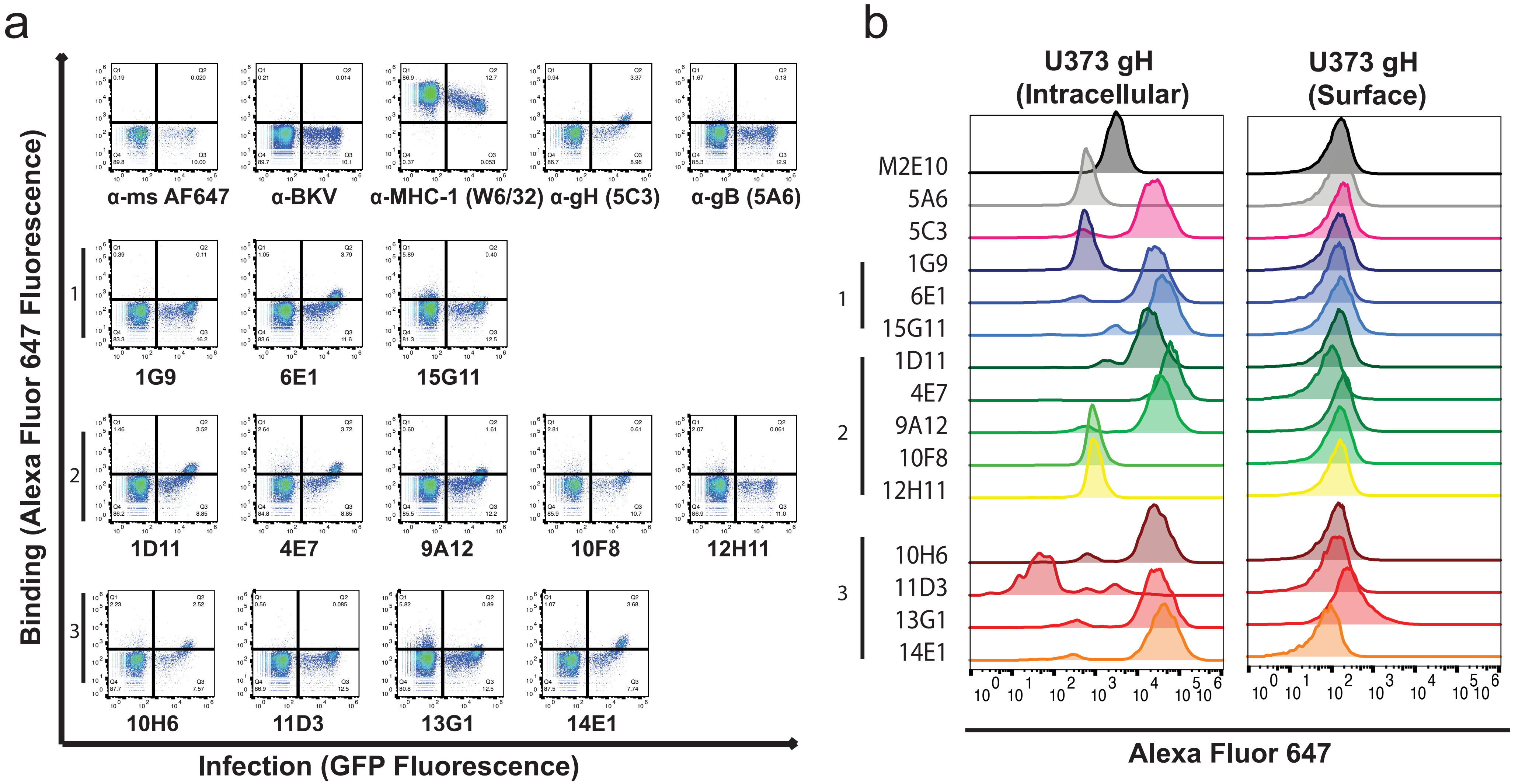

Supplementary Figure 5. The gH mAbs recognize conformational epitopes generated during HCMV infection. (a) ARPE-19 cells infected with AD169R (MOI 0.1) and collected 6 dpi were characterized for antibody (2 µg/mL) binding to the surface of infected cells by flow cytometry. (b) U373 gH cells were stained by immunofluorescence (2 µg/mL) and binding was quantified by flow cytometry. gH monomer does not traffic to the cell surface without complexing with gL or UL116 and thus no binding is detected in live cells. The majority of antibody are able to detect intracellular gH in fixed and permeabilized U373-gH cells. Each plot is a representative plot from technical triplicates.

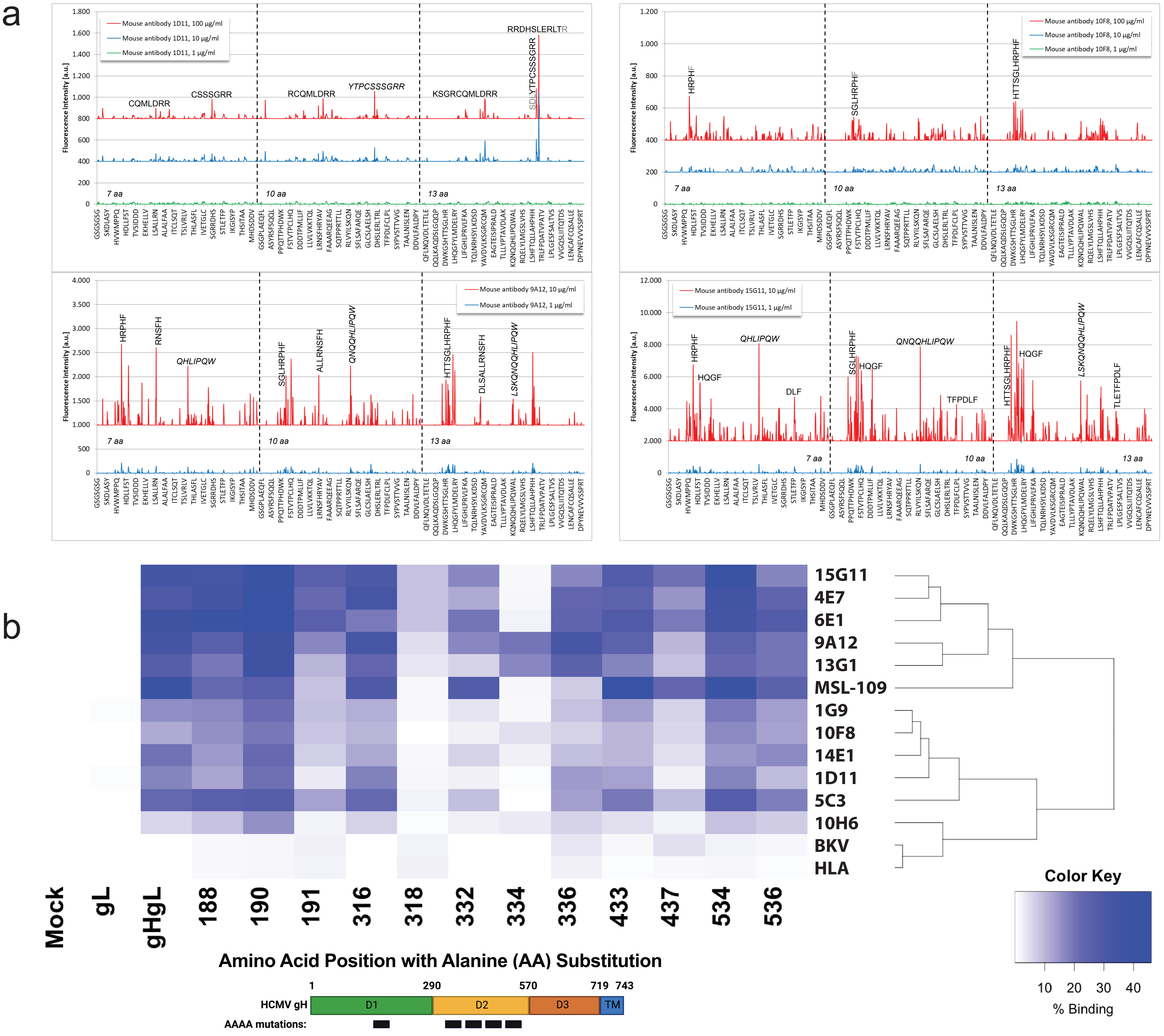

Supplementary Figure 6. Epitope mapping using gH peptide microarray and double alanine substitution. (a) The results for binding of 1D11, 10F8 and 5G11 to the overlapping peptide microarray using cyclic peptides of 7, 10 and 13 aa in length of the gH protein. (b) Heat map for binding profiles of labeled gH antibodies to gH AA mutants. The x axis denotes location of AA substitution and y axis is the percent binding to BHK cells transfected to express the gH AA mutants at 48h post co-transfection with gL. A schematic of each region subjected to AA mutation is provided below and indicated by black bars.

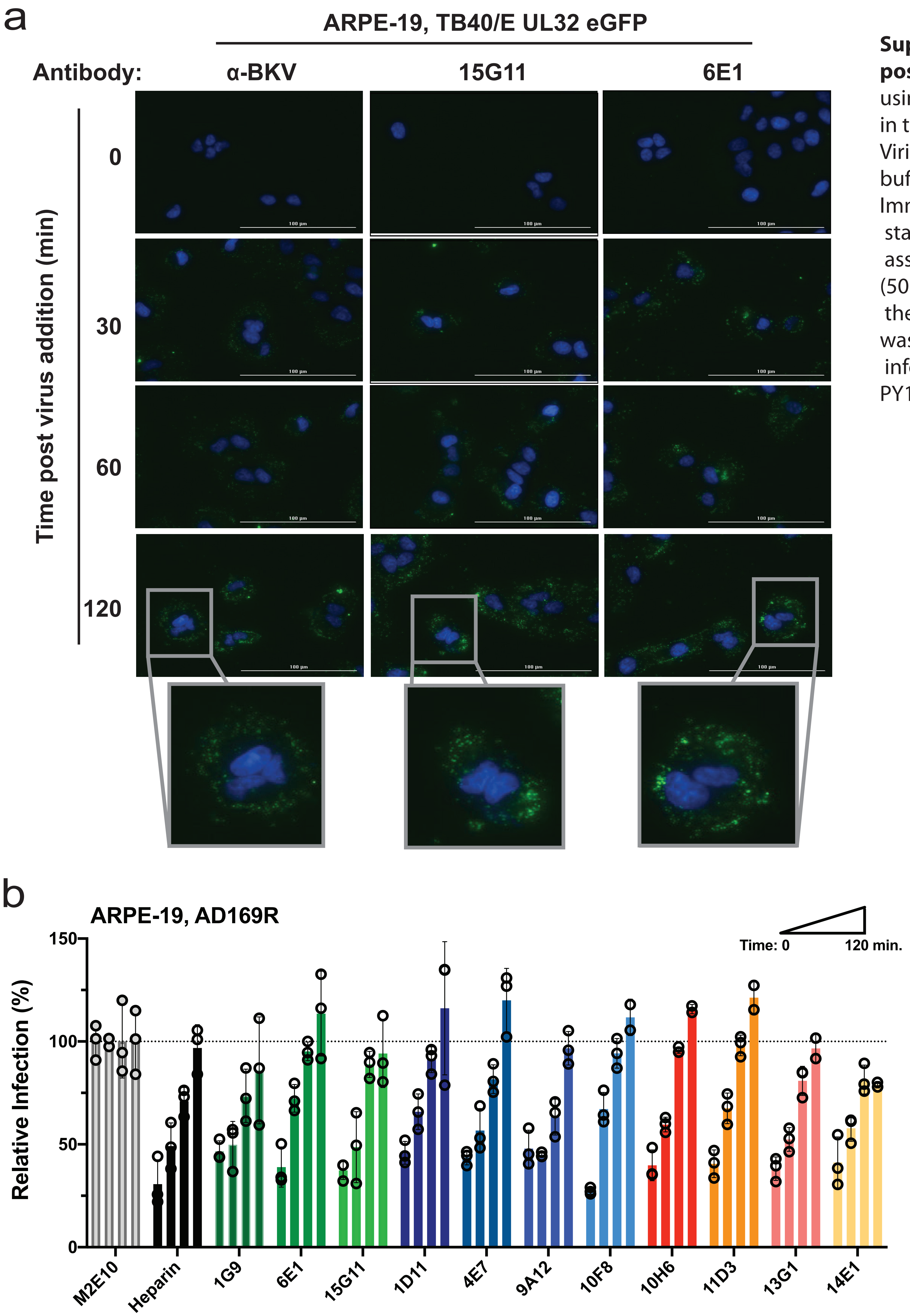

Supplementary Figure 7. α-gH antibodies limit infection at a post-attachment step. (a) An attachment assay was performed using ARPE-19 cells infected with TB40/E UL32-eGFP virus (green) in the presence of antibody; α-BKV, 15G11 or 6E1 (10 µg/mL). Virions present at the cell surface were removed using citric acid buffer (pH 3.5, 2 min) at 0, 30, 60 and 120 min after virus addition. Immunofluorescent images were taken on a Cytation 3 following stain with Hoechst reagent (0.01 µg/mL). (b) A time of addition assay was performed using ARPE-19 cells incubated with antibody (50 µg/mL) at 0, 30, 60, 120 min post virus addition (MOI 0.2) using the reporter virus AD169R. After 120 min both antibody and virus was removed and replaced with complete media. Relative percent infection was calculated at 18 hpi and the irrelevant antibody PY102 (α-IAV) used to normalize infection for each time point.

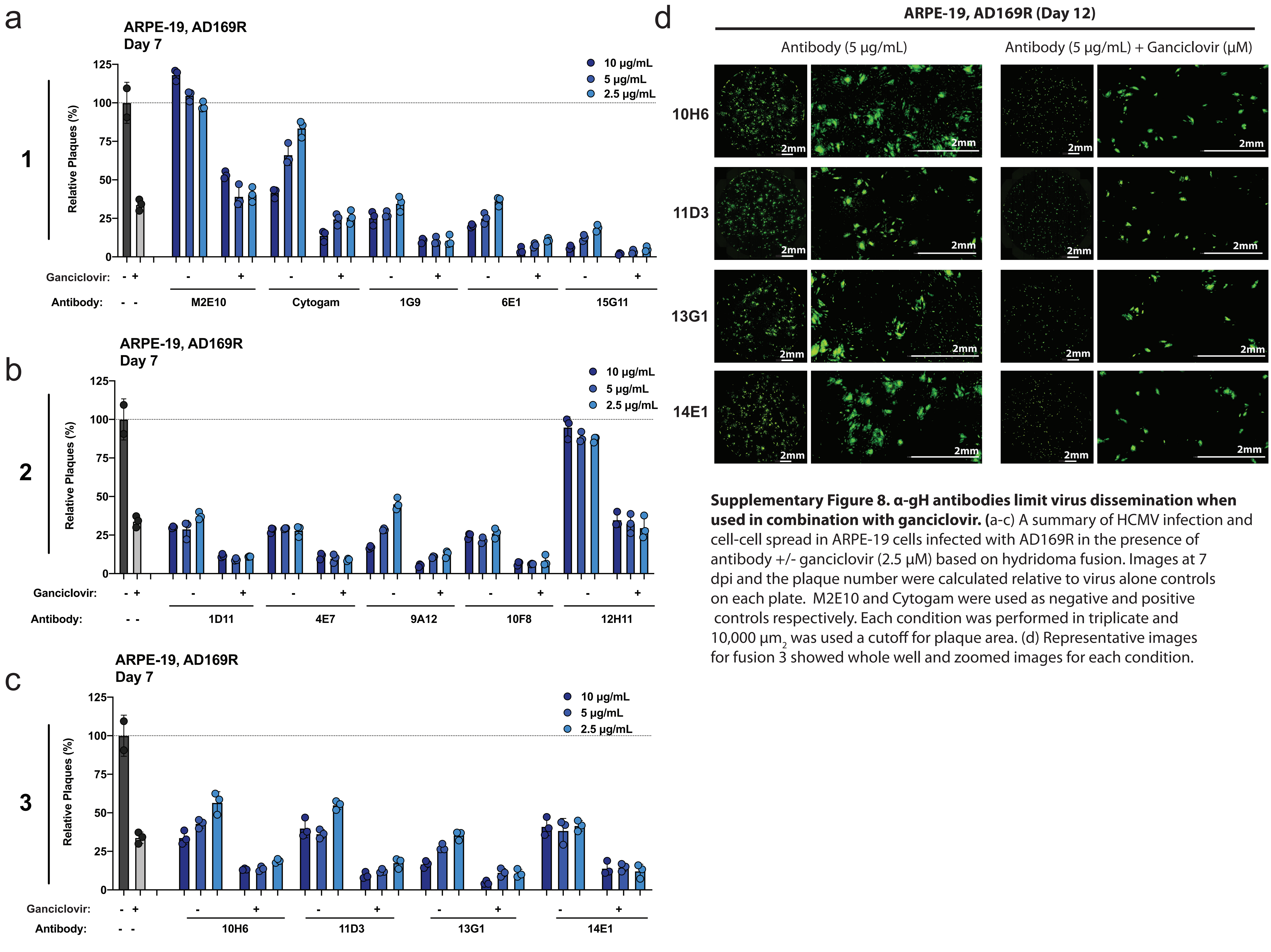

Supplementary Figure 8. α-gH antibodies limit virus dissemination when used in combination with ganciclovir. (a-c) A summary of HCMV infection and cell-cell spread in ARPE-19 cells infected with AD169R in the presence of antibody +/- ganciclovir (2.5 µM) based on hydridoma fusion. Images at 7 dpi and the plaque number were calculated relative to virus alone controls on each plate. M2E10 and Cytogam were used as negative and positive controls respectively. Each condition was performed in triplicate and 10,000 μm_2 was used a cutoff for plaque area. (d) Representative images for fusion 3 showed whole well and zoomed images for each condition.

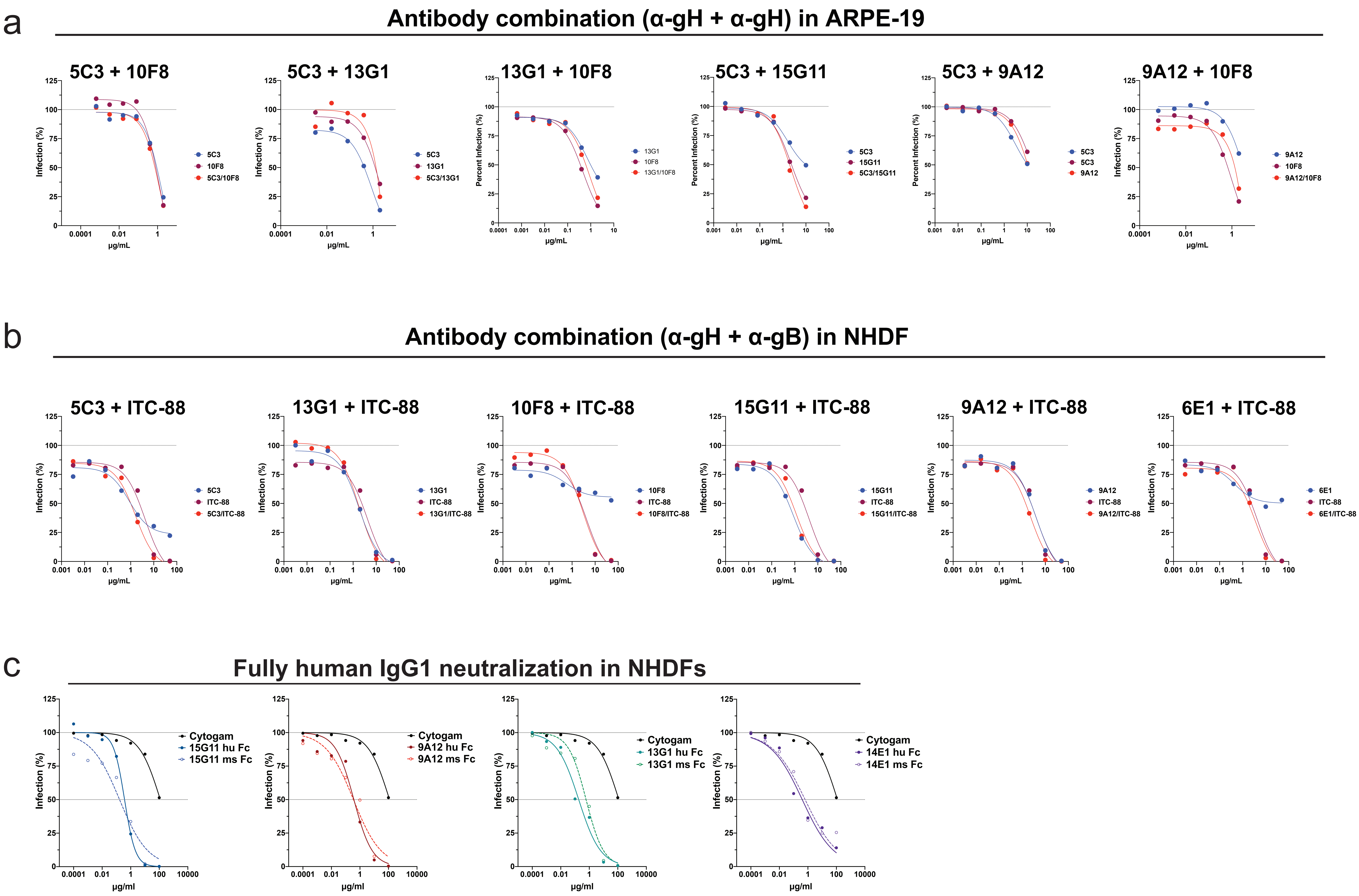

Supplementary Figure 9. Assessment of combination treatment for HCMV neutralization. (a) Neutralization in the presence of final antibody concentration (50-0.016 μg/mL) was assessed when two gH mAbs were used to neutralize AD169R (MOI 0.2) in ARPE-19 cells. (b) Neutralization curves generated after a gH antibody was used in combination with anti-gB neutralizing antibody ITC-88 for neutralization assay using NHDF cells. For all curves the combination treatment is shown in red and single antibody treatment is shown in blue and maroon. (c) Fully human IgG1 version of 15G11, 9A12, 13G1 and 14E1 mAbs were tested in parallel to hybrid versions of each antbody. Neutralization of AD169R (MOI 0.2) in NHDF fibroblasts was assessed at 18 hpi and normalized to virus only controls. All neutralization curves were generated using Log(inhibitor) vs response (3 parameters) equation was used to generate neutralization curves after the x-axis was transformed to a log scale using GraphPad Prism 9 software.

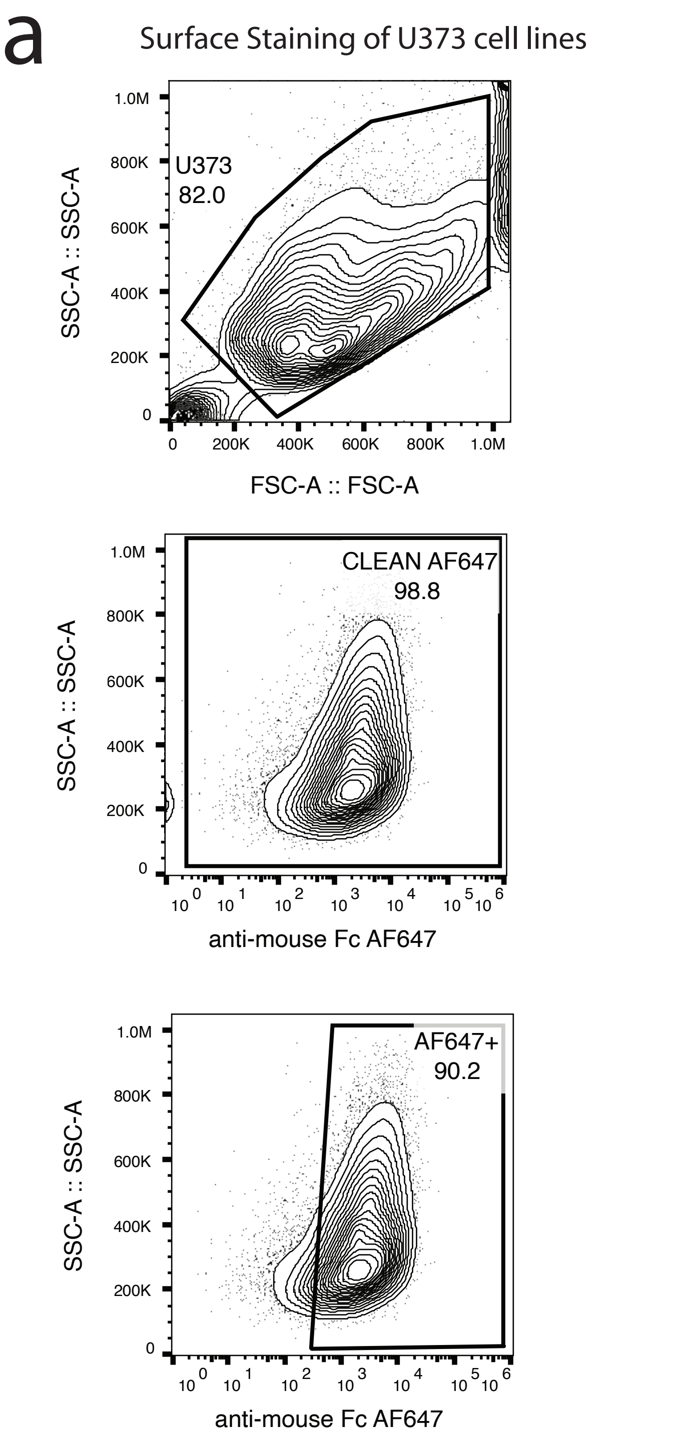

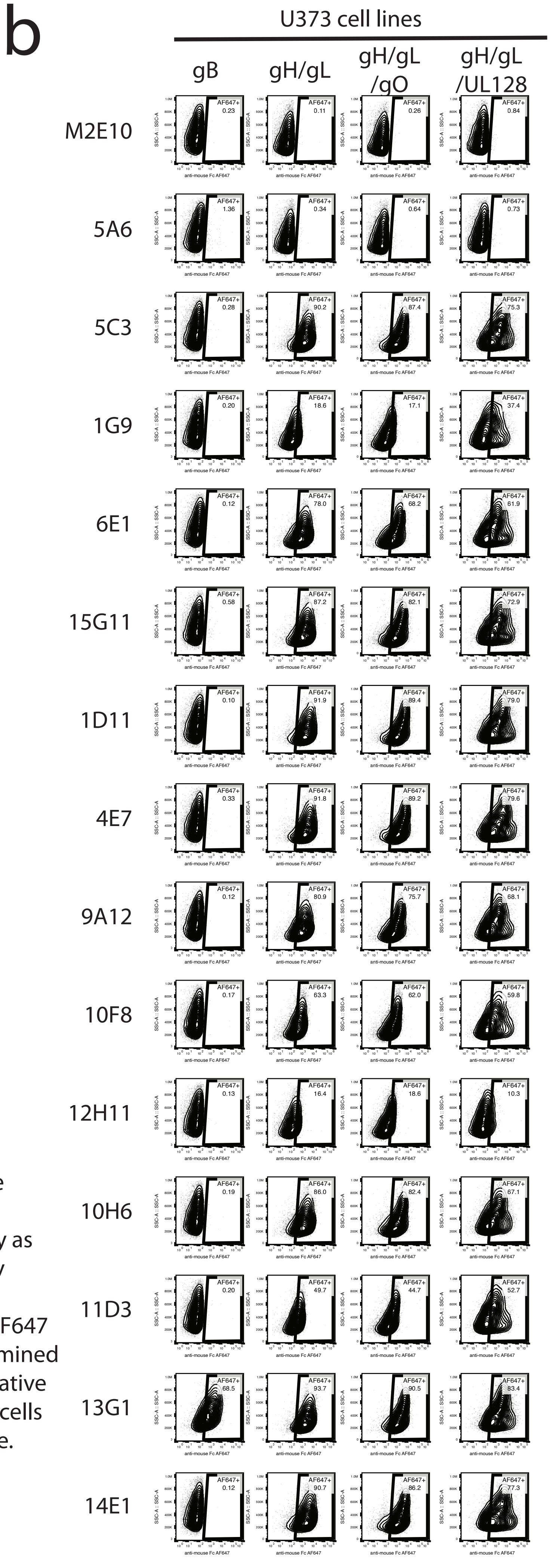

Supplementary Figure 10. Gating strategy and contour plots to accompany binding data for Figure 3b. (a) Live U373 cells expressing gH/gL were stained with the previously published gH antibody 5C3 (2 µg/mL) and then analyzed by flow cytometry as a positive control for gHgL expression and antibody binding. Samples were intially gated on FSC and SSC then cells below the limit of detection on the AF647 axis were removed. AF647 positive cells were determined using irrelevant antibody M2E10 (anti-IAV) as a negative control for binding. (B) A representation of AF647+ cells from each test condition are shown for each cell line. Each test condition was performed in triplicate. Proportion of AF647+ cells is listed as a percentage in each plot.

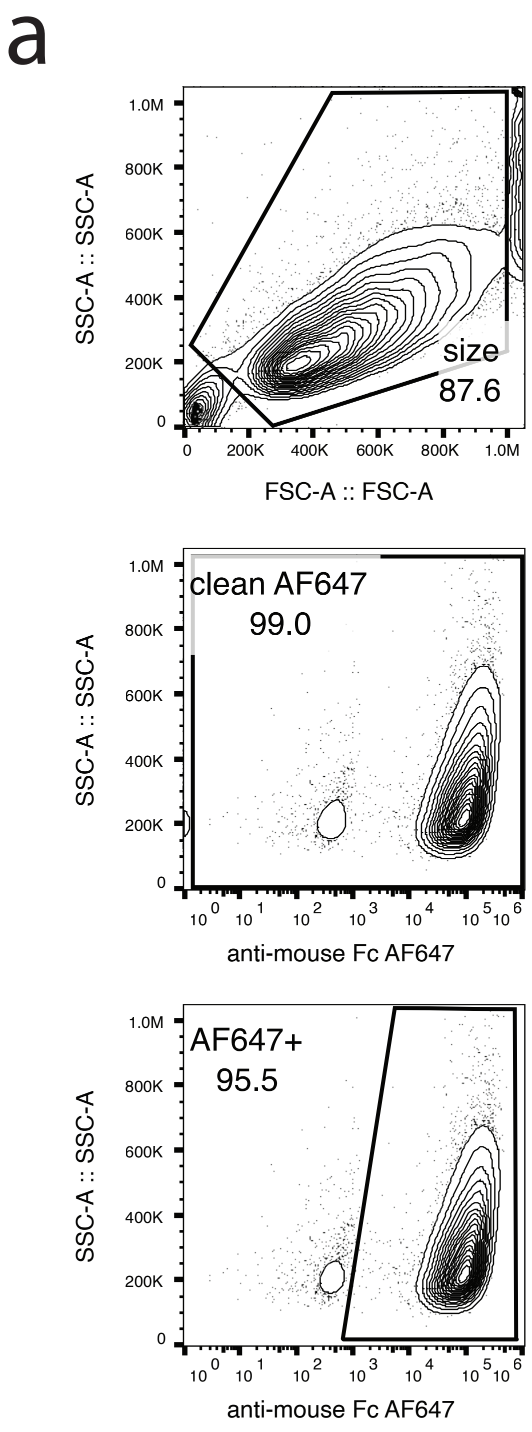

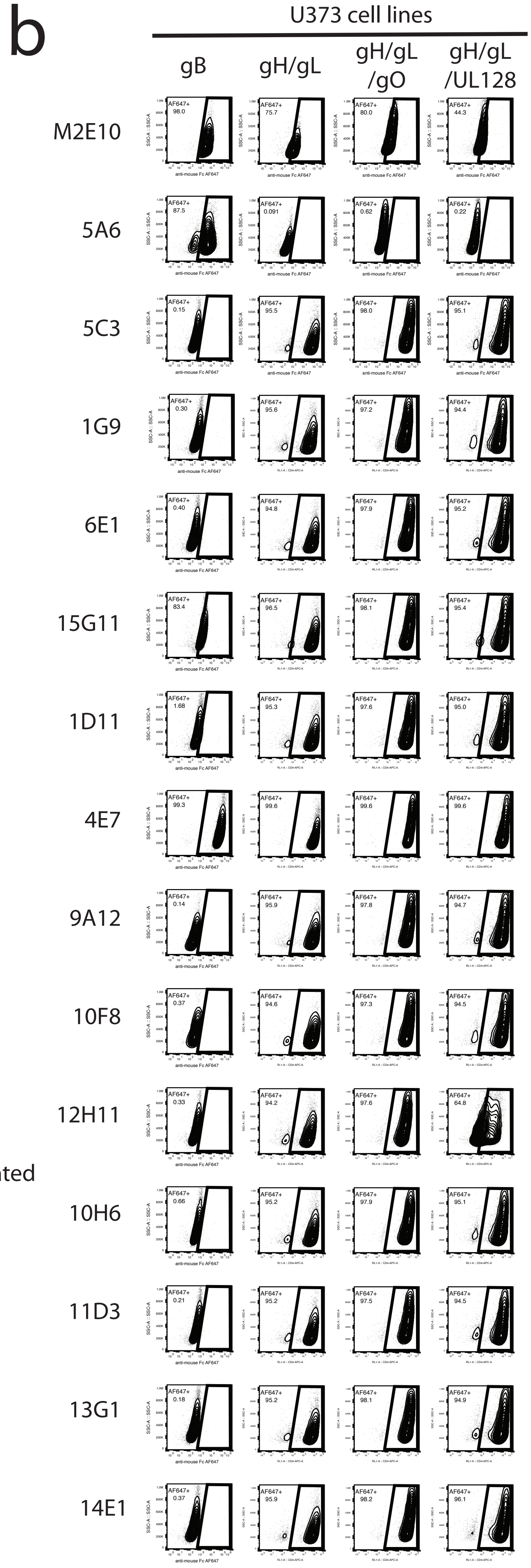

Supplementary Figure 11. Gating strategy and contour plots to accompany binding data from Figure 3b for each antibody (2 µg/mL) when incubated with fixed and permeabilized U373 astrocytoma cell lines. (a) Representative gating strategy showing U373-gH/gL cells stained with 5C3, a previously characterized gH monoclonal, as a positive control for gH expression and binding. (b) A representative contour plot of AF647+ cells from each test condition are shown for each cell line. All conditions were performed in triplicate. Proportion of AF647+ cells are listed for each plot.

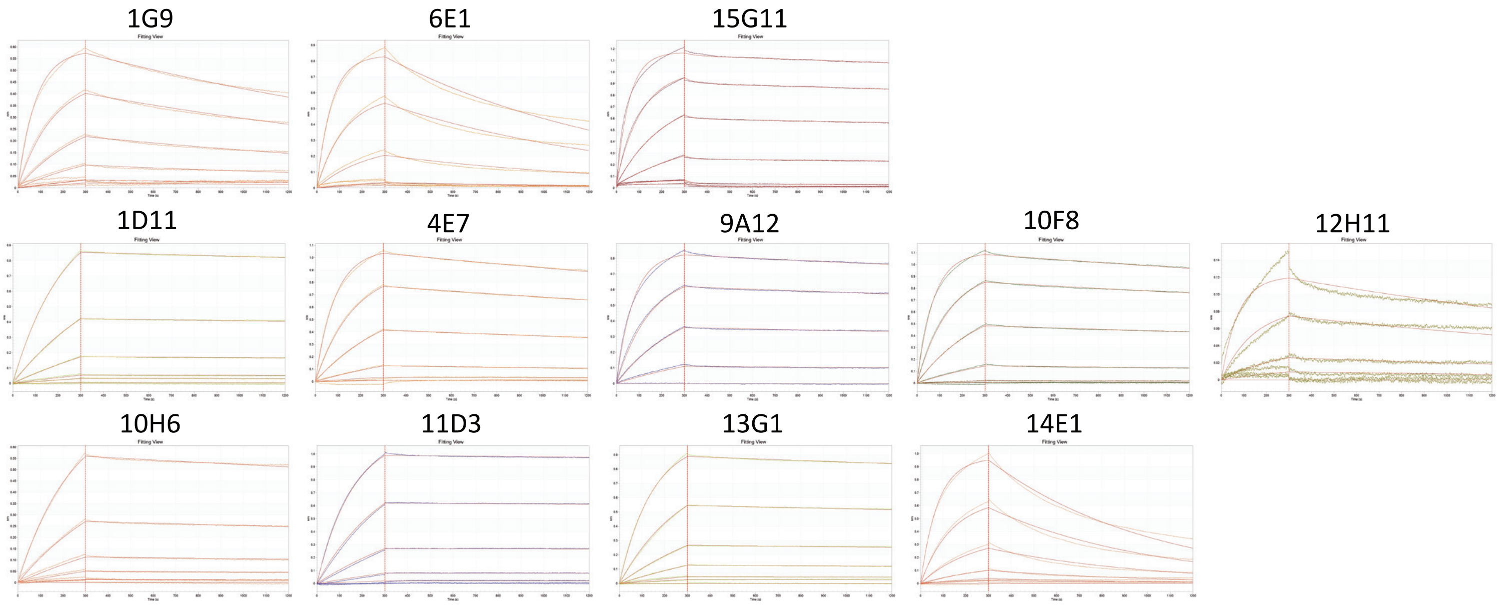

Supplementary Figure 12. Binding sensograms of monoclonal antibodies: Octet sensograms of binding experiments using recombinant gH/gL/Ul128/Ul130/Ul131a pentamer complex (rPC) at concentrations listed. Serial two fold dilutions of rPC were made and incubated with capture sensors pre-incubated with 10ug of antibody for each antibody listed. Interactions between immobilized mouse Fc antibodies and recombinant pentamer are shown. Y axis represents binding and X axis represents time (s). Following attachment, dissociation was performed in PBS. 1G9 (540.5, 270.3, 135.1, 67.6, 33.8, 16.9, 8.45nM); 6E1 (270.3, 135.1, 67.6, 33.8, 16.9nM), 15G11 (540.5, 270.3, 135.1, 67.6, 33.8, 16.9nM), 1D11 (540.5, 270.3, 135.1, 67.6, 33.8, 16.9nM), 4E7 (540.5, 270.3, 135.1, 67.6, 33.8nM), 9A12 (540.5, 270.3, 135.1, 67.6nM), 10F8 (540.5, 270.3, 135.1, 67.6, 33.8nM), 12H11 (540.5, 270.3, 135.1, 67.6, 33.8, 16.9nM), 10H6 (540.5, 270.3, 135.1, 67.6, 33.8, 16.9, 8.45nM), 11D3 (540.5, 270.3, 135.1, 67.6, 33.8nM) , 13G1 (540.5, 270.3, 135.1, 67.6, 33.8, 16.9, 8.45nM); 14E1 (270.3, 135.1, 67.6, 33.8, 16.9nM).

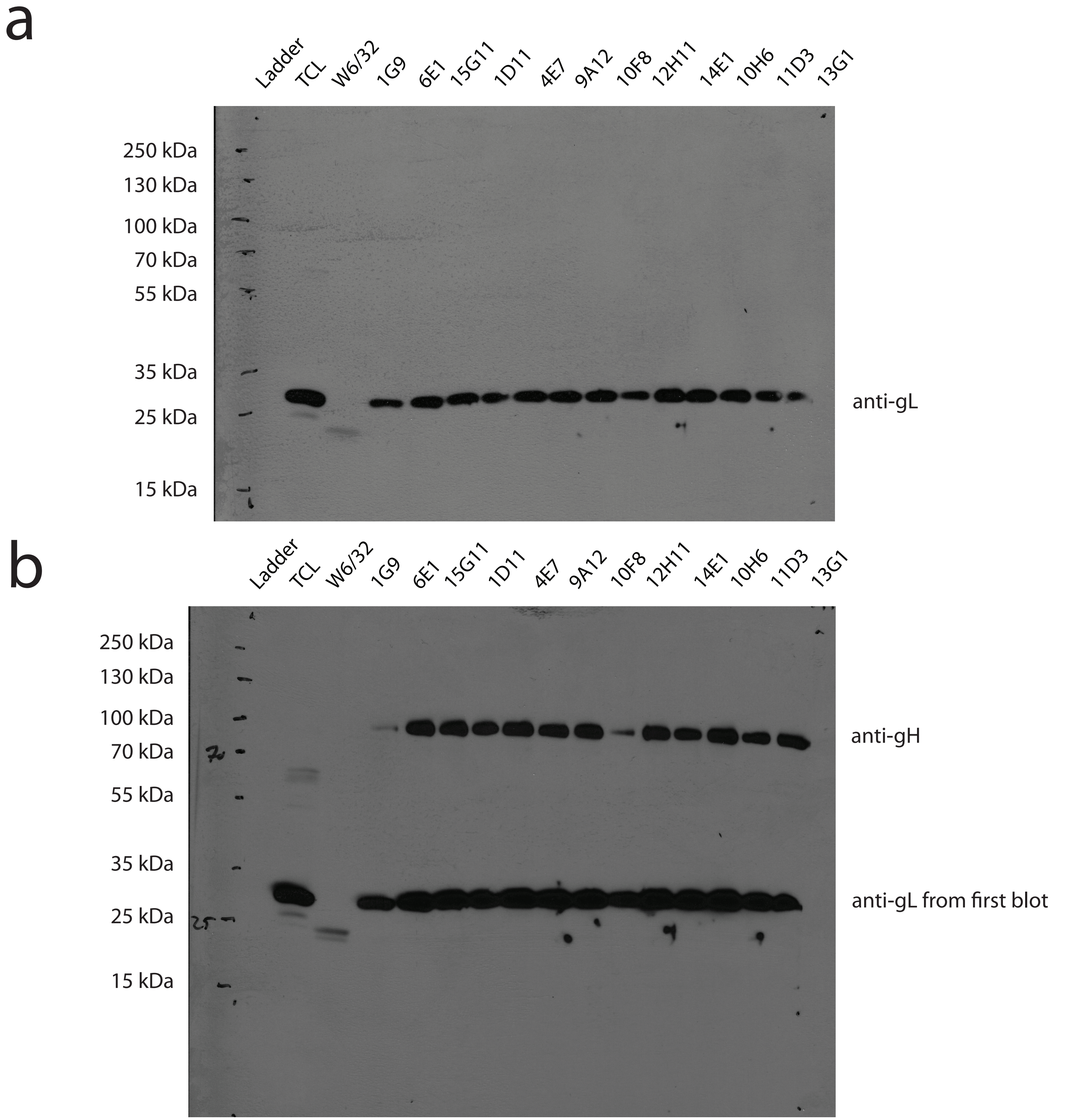

Supplementary Figure 13. Raw blot data related to Figure 3C. (a) Unprocessed western blot scans related to immunoprecipitation of U373 gH/gL total cell lysates (TCL) with antibodies indicated above each lane to confirm gH/gL binding. Membrane was initially blotted with polyclonal rabbit antibodies raised against gL peptide. Anti-rabbit IgG-HRP detection antibody was used to detect primary antibody. (b) Unproccessed scan of the same western blot after probing with polyclonal rabbit anti-gH antibody and detecting using anti-rabbit IgG HRP detection antibody. Dots at the edges of each corner were made to indicate the cormers of the PVDF membrane on the X-ray film after chemiluminescent development. The ProteinPlus ladder loaded in the first lane is outlined in permament marker to track size of each marker. TCL of U373-gHgL cells was used as a positive loading control for gHgL expression.

Supplementary Table 1.

Antibody	IC$_{50}$ (ug/mL) hu Fc IgG1	ms Fc IgG2a
15G11	0.37	0.17
9A12	0.37	0.35
13G1	0.18	0.60
14E1	0.40	0.64

Supplementary Table 1. Half-maximal inhibitory concentration (IC$_{50}$) for fully human mAbs after mouse Fc regions were replaced. The IC$_{50}$ for newly produced fully human antibodies is listed. NHDF fibroblasts were infected with AD169R (MOI 0.2) pre-incubated with antibody diluted from 100 μg/mL using 10-fold dilutions.
