# Supplementary material for: Development of broadly neutralizing antibodies targeting the cytomegalovirus subdominant antigen gH
Source: Commun Biol. 2022 Apr 25;5:387. doi: 10.1038/s42003-022-03294-z (PMC9038728; doi:10.1038/s42003-022-03294-z)
Supplement: Supplementary file 3 — Reporting Summary [file 42003_2022_3294_MOESM3_ESM.pdf]

nature portfolio

Corresponding author(s): DBPR COMMSBIO-21-2636-A
Last updated by author(s): Feb 11, 2022

Reporting Summary

Nature Portfolio wishes to improve the reproducibility of the work that we publish. This form provides structure for consistency and transparency in reporting. For further information on Nature Portfolio policies, see our Editorial Policies and the Editorial Policy Checklist.

Statistics

For all statistical analyses, confirm that the following items are present in the figure legend, table legend, main text, or Methods section.

n/a	Confirmed	
☐	☒	The exact sample size (n) for each experimental group/condition, given as a discrete number and unit of measurement
☐	☒	A statement on whether measurements were taken from distinct samples or whether the same sample was measured repeatedly
☐	☒	The statistical test(s) used AND whether they are one- or two-sided<br>*Only common tests should be described solely by name; describe more complex techniques in the Methods section.*
☒	☐	A description of all covariates tested
☐	☒	A description of any assumptions or corrections, such as tests of normality and adjustment for multiple comparisons
☐	☒	A full description of the statistical parameters including central tendency (e.g. means) or other basic estimates (e.g. regression coefficient) AND variation (e.g. standard deviation) or associated estimates of uncertainty (e.g. confidence intervals)
☒	☐	For null hypothesis testing, the test statistic (e.g. F, t, r) with confidence intervals, effect sizes, degrees of freedom and P value noted<br>*Give P values as exact values whenever suitable.*
☒	☐	For Bayesian analysis, information on the choice of priors and Markov chain Monte Carlo settings
☒	☐	For hierarchical and complex designs, identification of the appropriate level for tests and full reporting of outcomes
☒	☐	Estimates of effect sizes (e.g. Cohen's d, Pearson's r), indicating how they were calculated

Our web collection on statistics for biologists contains articles on many of the points above.

Software and code

Policy information about availability of computer code

Data collection	Nexcelom Celigo, Intellicyte HTFC, and Attune Cytometer built-in software
Data analysis	GraphPad, FlowJo

For manuscripts utilizing custom algorithms or software that are central to the research but not yet described in published literature, software must be made available to editors and reviewers. We strongly encourage code deposition in a community repository (e.g. GitHub). See the Nature Portfolio guidelines for submitting code & software for further information.

Data

Policy information about availability of data

All manuscripts must include a data availability statement. This statement should provide the following information, where applicable:

- Accession codes, unique identifiers, or web links for publicly available datasets
- A description of any restrictions on data availability
- For clinical datasets or third party data, please ensure that the statement adheres to our policy

The primary data will become available upon request and for research purposes only.

Field-specific reporting

Please select the one below that is the best fit for your research. If you are not sure, read the appropriate sections before making your selection.

☒ Life sciences ☐ Behavioural & social sciences ☐ Ecological, evolutionary & environmental sciences

For a reference copy of the document with all sections, see nature.com/documents/nr-reporting-summary-flat.pdf

Life sciences study design

All studies must disclose on these points even when the disclosure is negative.

Sample size	The sample size for each experiment was included in the Methods, Results, or Figure Legend.
Data exclusions	No data was excluded from the figures
Replication	The experimental findings were replicated at least 2 times and in some cases three times.
Randomization	The samples were not randomized because of the experimental setup.
Blinding	The analysis of experiments was measured in an unbiased manner and the data was collected and displayed as the respective intensities.

Reporting for specific materials, systems and methods

We require information from authors about some types of materials, experimental systems and methods used in many studies. Here, indicate whether each material, system or method listed is relevant to your study. If you are not sure if a list item applies to your research, read the appropriate section before selecting a response.

Materials & experimental systems

n/a	Involved in the study	
☐	☒	Antibodies
☐	☒	Eukaryotic cell lines
☒	☐	Palaeontology and archaeology
☒	☐	Animals and other organisms
☒	☐	Human research participants
☒	☐	Clinical data
☒	☐	Dual use research of concern

Methods

n/a	Involved in the study	
☒	☐	ChIP-seq
☐	☒	Flow cytometry
☒	☐	MRI-based neuroimaging

Antibodies

Antibodies used	The antibodies in this study are listed with the requested information within the Material and Methods of the manuscript.
Validation	The validation of the antibodies was provided in previous and current manuscripts.

Eukaryotic cell lines

Policy information about cell lines

Cell line source(s)	The cell lines were purchased from ATCC.
Authentication	The cell lines were not authenticated.
Mycoplasma contamination	Cell lines used in the study tested negative for mycoplasma.
Commonly misidentified lines (See ICLAC register)	None

Flow Cytometry

Plots

Confirm that:

☒ The axis labels state the marker and fluorochrome used (e.g. CD4-FITC).

☒ The axis scales are clearly visible. Include numbers along axes only for bottom left plot of group (a 'group' is an analysis of identical markers).

☒ All plots are contour plots with outliers or pseudocolor plots.

☒ A numerical value for number of cells or percentage (with statistics) is provided.

Methodology

Sample preparation

Figure 3A: The ARPE-19 epithelial cell line was infected in vitro using AD169R reporter virus which expresses GFP and cells were harvested for flow analysis on day 7 post infection in parallel with uninfected ARPE-19 cells.
For intracellular staining (Figure 3A-B) Cells were trypsinized (0.05% trypsin) to obtain single cell suspension then trypsin was neutralized with complete media containing 10% fetal bovine serum. Cells were pelleted at 1500 rpm for 5 min at 4C and then washed with PBS before being fixed and permeabilized (20 minutes, 4C) for staining (Cytofix/CytoPerm, BD Biosciences). The staining buffer for all steps post-fix was 1% BSA/0.1% Saponin in PBS and fixed cells were spun at 1800 rpm for 5 minutes to pellet in each subsequent step. Fixed cells were plated in a 96 well plate and stained using 2 ug/mL of mouse monoclonal antibodies for 1 hr at 4C before washing two times staining buffer and adding secondary antibody (goat anti-mouse Fc AF647, 4 ug/mL final conc.). Stained cells were washed twice with staining buffer and resuspended in a final volume of 100uL for sample acquisition.
For surface staining, live trypsinized cells in a single cell suspension were washed with PBS and stained in buffer containing 1% BSA/PBS. All antibodies and antibody concentrations were kept consistent as explained above (2 ug/mL primary antibody and 4ug/mL secondary antibody) with washes between primary and secondary staining steps.

Instrument

Figure 3A: Intellicyte HTFC Screening System Flow Cytometer; Figure 3B: Attune NxT Acoustic Focusing Cytometer

Software

The software within the HTFC Screening system is ForeCyt acquisition and analysis software. FCS files were exported to analyze and graph data using FlowJo version 10.2.

Cell population abundance

Figure 3A: ~98% of the cells collected on day 7 post infection were infected based on GFP expression and binding by gH maBs ranged from 2.32% (isotype negative control) to 86.3% (W6/32 positive control) based on AF647 secondary antibody expression. ~60% of mock and 50% of infected cells were included in downstream analysis after initial gating on FSC-A vs SSC-A for size and viability.
Figure 3B: The abundance of the U373 astrocytoma cell lines which constitutively express the HCMV glycoproteins (gB, gH/gL, gH/gL/gO, gH/gL/UL128) varied slightly based on cell line but typically ran between 10,000-15,000 cells (~75% of total cells collected) after gating on FSC-A vs SSC-A to remove debris and atypical cell sizes (25%). The gHgL cells had slightly higher viability (88%) after gating on size.

Gating strategy

Figure 3A:
1. FSC-A vs SSC-A
2. FL1-GFP vs FL4-AF647
Cells were first gated based on size (FSC-A vs SSC-A) then a quadrant plot was generated with FL1-GFP as a readout for infection on the x axis an antibody binding (FL4-AF647) on the y axis (FL1-A vs FL4-A). Uninfected cells for each condition were overlaid on top of the quadrant plot of infected cells stained with the same antibody in order to compare relative binding to uninfected cells. The boundary for GFP was placed based on position of uninfected cells and the boundary for AF647 was based on the average AF647 values for mock samples stained with different antibodies within the panel.
Figure 3B:
1. FSC-A vs SSC-A size
2. SSC-A vs RL1-A (AF647 laser channel, removes events along x-axis (low MFI)
3. SSC-A vs RL1-A+ gates on AF647+ cells

☒ Tick this box to confirm that a figure exemplifying the gating strategy is provided in the Supplementary Information.
